# Antibiofilm activity substances derived from coral symbiotic bacterial extract inhibit biofouling by the model strain *Pseudomonas aeruginosa* PAO1

**DOI:** 10.1111/1751-7915.13312

**Published:** 2018-10-09

**Authors:** Yu Song, Zhong‐Hua Cai, Yong‐Min Lao, Hui Jin, Ke‐Zhen Ying, Guang‐Hui Lin, Jin Zhou

**Affiliations:** ^1^ Department of Earth System Science Tsinghua University of Education Key Laboratory for Earth System Modeling Beijing 100084 China; ^2^ Division of Ocean Science and Technology Graduate School at Shenzhen Tsinghua University Shenzhen 518055 China

## Abstract

The mitigation of biofouling has received significant research attention, with particular focus on non‐toxic and sustainable strategies. Here, we investigated quorum sensing inhibitor (QSI) bacteria as a means of controlling biofouling in a laboratory‐scale system. Approximately, 200 strains were isolated from coral (*Pocillopora damicornis*) and screened for their ability to inhibit quorum sensing (QS). Approximately, 15% of the isolates exhibited QSI activity, and a typical coral symbiotic bacterium, H12‐*Vibrio alginolyticus*, was selected in order for us to investigate quorum sensing inhibitory activity further. Confocal microscopy revealed that *V. alginolyticus* extract inhibited biofilm formation from *Pseudomonas aeruginosa* PAO1. In addition, the secondary metabolites of *V. alginolyticus* inhibited PAO1 virulence phenotypes by downregulating motility ability, elastase activity and rhamnolipid production. NMR and MS spectrometry suggested that the potential bioactive compound involved was rhodamine isothiocyanate. Quantitative real‐time PCR indicated that the bacterial extract induced a significant downregulation of QS regulatory genes (*lasB, lasI, lasR, rhlI, rhlR*) and virulence‐related genes (*pqsA*,* pqsR*). The possible mechanism underlying the action of rhodamine isothiocyanate analogue involves the disruption of the *las* and/or *rhl* system of PAO1. Our results highlight coral microbes as a bioresource pool for developing QS inhibitors and identifying novel antifouling agents.

## Introduction

Marine biofouling, defined as the rapid and extensive growth of marine organisms on submerged inanimate and living surfaces, is a severe problem worldwide. The establishment of biofilms in the aquatic environment typically begins with the settlement of microorganisms (bacteria and unicellular eukaryotes), followed by the recruitment of macrofouling species (invertebrate larvae and algal spores) (Dobretsov, 2009) whose accumulation leads to considerable economic consequences for aquaculture equipment, naval vessels and a variety of industrial structures (Braithwaite and McEvoy, 2005; Schultz *et al*., 2011; Fitridge *et al*., 2012). Although biocide treatments, including antifouling paints (such as tributyltin and copper), are effective against biofouling, many of these toxins are highly destructive pollutants when used in marine ecosystems (Voulvoulis *et al*., 2002; Yebra *et al*., 2004; Thomas and Brooks, 2010). Consequently, there is a need to pursue novel, environmental‐friendly, and non‐toxic compounds that are capable of inhibiting the development of marine biofilms.

The fouling process is often thought of as a general sequence of colonization in terms of settlement and the establishment of a complex community, with the irreversible attachment of bacteria and the formation of a complex film‐like structure as the two key initial steps. The latter are being increasingly recognized for their important roles in the fouling process, and inhibitors of biofilm formation or interference with bacterial signalling processes can mitigate the development of biofilm or even lead to biofilm decline without any toxic effects on the microbes (Deziel *et al*., 2001). For example, nitric oxide (NO), an intracellular signalling molecule, has been shown to induce a reduction in biofilm (Barraud *et al*., 2006).

Another more important aspect of signalling is quorum sensing (QS), which regulates the formation and maturation of biofilm during its development (Labbate *et al*., 2004). Research targeting the bacterial QS system has paid significant attention to the identification of effective QS or biofilm inhibition agents. Therefore, using QS inhibitors or QSI compounds that degrade signalling molecules to mitigate biofilm fouling represents a significant breakthrough (Dobretsov, 2009). Since then, several studies have demonstrated that QSI compounds are capable of reducing the formation of biofilm and further inhibit the development of biofouling (Cushnie and Lamb, 2011; Dobretsov *et al*., 2011; Karina *et al*., 2013). Some antifouling substances have recently been identified in microbes and plants (and even animals); these are peptide compounds, such as quorum‐quenching enzyme and small‐molecule compounds (Huang *et al*., 2016; Bzdrenga *et al*., 2017). Of particular note, is furanone, a traditional inhibitor that is a potent antagonist of Gram‐negative bacteria (Hentzer *et al*., 2003), and a type of long‐chain fatty aldehyde, identified as pentadecanal, which acts against *Staphylococcus epidermidis* biofilm (Casillo *et al*., 2017), as well as the 2‐sufonylpyrimidines which can significantly inhibit *Pseudomonas aeruginosa* biofilm (Thomann *et al*., 2016).

The marine environment is a rich storehouse of natural products possessing antibiofilm properties, and accumulating evidence has confirmed the existence of a diverse array of active compounds in coral, bryozoans, sponge and pyrosomida (Skindersoe *et al*., 2008; Tello *et al*., 2012). Furthermore, studies increasingly point to types of planktonic marine bacteria, such as *Bacillus* spp., *Pseudomonas* spp., *Pseudoalteromonsa* spp., and *Vibrio* spp., as frequent sources for the production of QSI compounds (Teasdale *et al*., 2009; Mangwani *et al*., 2015; Benneche *et al*., 2016; Casillo *et al*., 2017). Compared with the planktonic environment, coral ecosystems have a much higher biodiversity and represent a potential source from which to isolate QS inhibitors. Indeed, several studies have described the QSI or antibiofilm potential of several natural compounds originating from coral, including the soft coral *Antillogorgia elisabethae*, the black coral *Antipathes dichotoma*, the octocoral *Eunicea knighti* and the cave coral *Acropora millepora* (Tello *et al*., 2012; Karina *et al*., 2013; Diana *et al*., 2016). The wide existence of QSI bacteria in coral environments invites us to explore the feasibility of QSI‐based biofouling control methods. We hypothesize that coral‐associated bacteria, and their metabolic substances, offer an effective antibiofouling resource in the marine environment.

Despite the abundance of bioactive compounds originating from coral symbiotic organisms, detailed information on the identification of these compounds has yet to be described (Bakkiyaraj *et al*., 2013). This present study emphasizes the importance of coral‐associated bacteria as a potential model for obtaining naturally occurring products with QSI properties. Given the limited knowledge available on their production by coral bacteria, the aim of our study was to gain a clearer understanding of the ecological relevance of QSI substances secreted by coral‐symbiotic microbes. Here, we used coral as the source material to screen QSI bacteria, and explored isolated bacteria for their QSI potential. Importantly, the bioactive compounds from these bacteria were identified, the expression levels of functional genes were analysed, and the possible antifouling mechanism of action was inferred.

## Results

### Isolation and identification of QSI bacteria from coral

**Figure 1:**
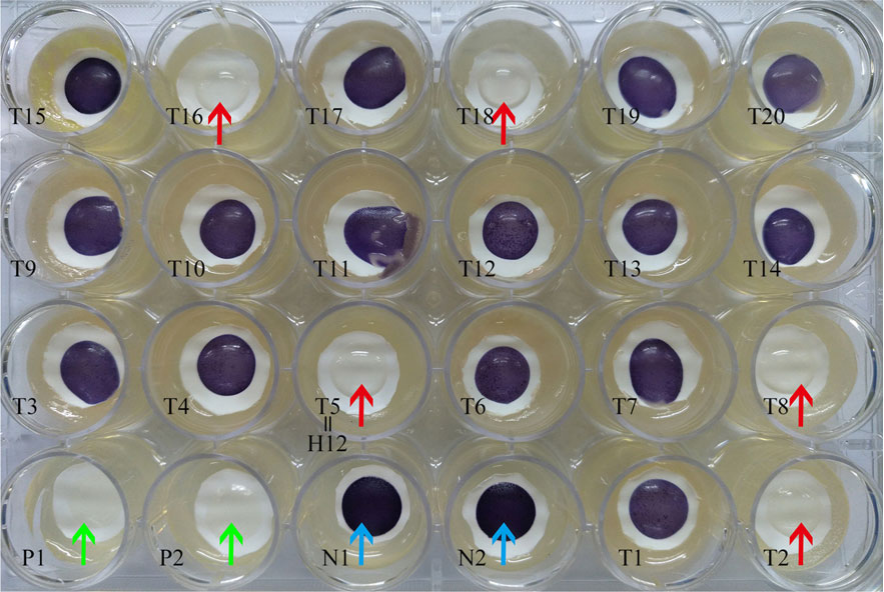
Screening of QSI strains on a 24‐well plate. The plate contained the biosensor strain, *C. violaceum* 12472 and filter paper with an aperture of 0.22 μm was used for sample detection. LB medium (blue arrows) and furanone (green arrows) served as negative and positive controls respectively. The absence of purple, or the formation of pigment inhibition, was considered indicative of potential QSI positive isolates. The test samples (red arrows, T2, T5, T8, T16 and T18) point to the positive QSI strains and pigment inhibition can be observed on a clear background on the plate. P1 & P2, as well as N1 & N2, represent the two positive controls and two negative controls respectively. T1 to T20 represent the test samples; T5 is the strain H12 used in this study.

We screened a total of 30 potential QSI isolates, which all caused colour reduction in *Chromobacterium violaceum* ATTC12472 (Table S1); representative results are shown in Fig. 1. Some isolates showed promising QSI activity and a clear inhibition of pigment was observed in the 24‐well plate containing the reference strain *C. violaceum* 12472; in contrast, the zone of inhibition was not detected with the negative control (LB medium) (Fig. 1). The activity of positive isolates was recorded as either strong (+++), medium (++), or weak (+) based on the extent of visible colourlessness by the biosensor (Table S1). The isolate H12 caused a relatively significant reduction, in that the purple pigment of *C. violaceum* ATTC12472 was completely eliminated (Fig. 1).

Most of the representative isolates shared a 99% 16s RNA sequence similarity with their respective reference strains. After filtering out the low‐quality sequences, and any duplicates from the thirty positive isolates, five representative QSI strains (*Vibrio alginolyticus, Staphylococcus hominis, Lysinibacillus fusiform, Staphylococcus warneri* and *Bacillus cereus*) were screened. Their bacterial sequences were submitted to the GenBank Database under the accession numbers MG761744–MG761748. Of the five strains, the isolated strain H12 caused significant colourlessness; this strain had 100% sequence similarity to *V. alginolyticus* (GenBank number JX566662.1). We tentatively described this as *V. alginolyticus* 12. Since this was the most active QSI agent, we focused on *V. alginolyticus* 12 for all subsequent experiments.

### Growth and violacein production

**Figure 2:**
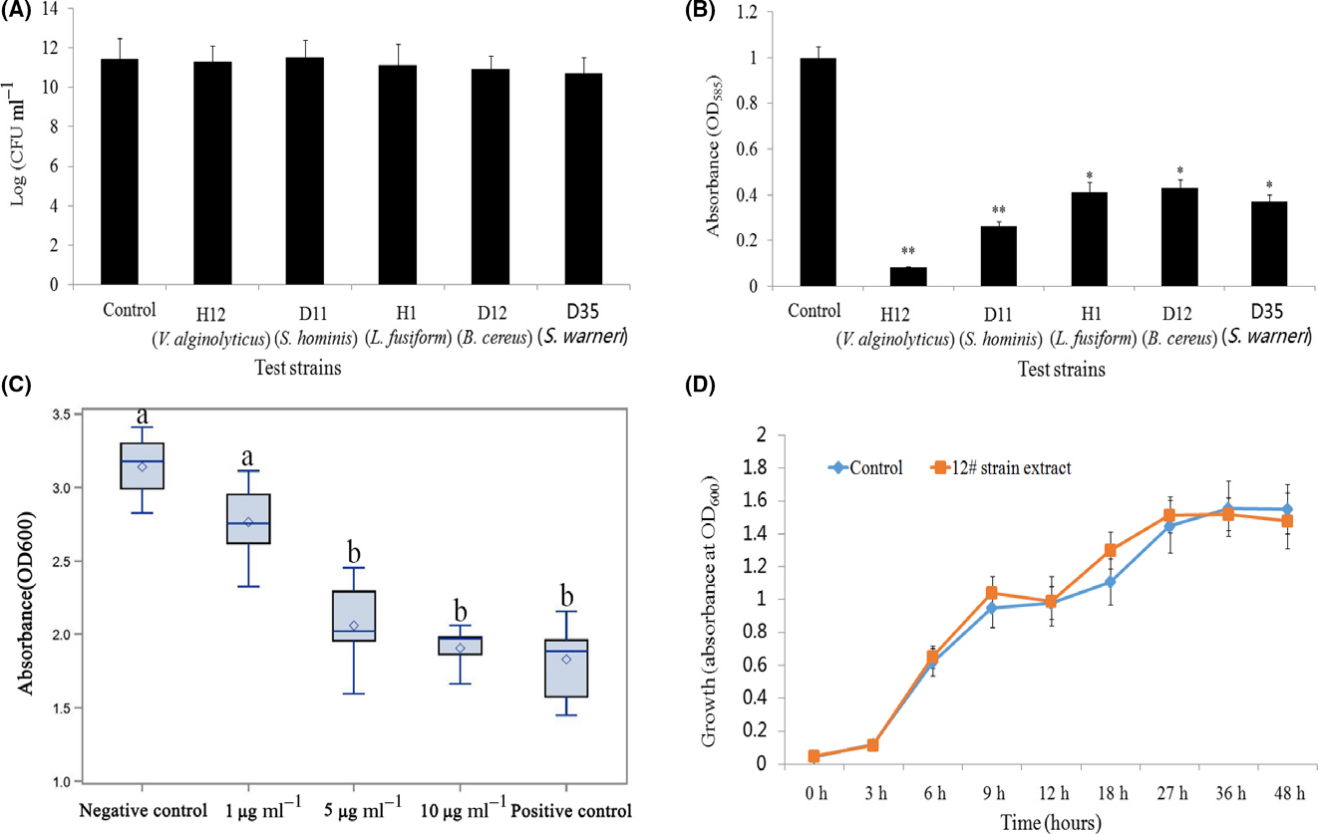
Effect on the colony growth of *C. violaceum* 12472. A. Bacterial cell counts from the flask incubation assay. The five tested strains were H12 (*V. alginolyticus*), D11 (*S. hominis*), H1 (*L. fusiform*), D12 (*B. cereus*) and D35 (*S. warneri*). CV12472 was incubated for 16 h, with 100 μl of the bacteria, adjusted to an OD_600 nm_ of 0.1, spread onto the LB plates. Bacterial growth inhibition was compared with corresponding controls. Data are shown as the logarithm of mean CFU ml^−1^ ± SD. B. Inhibition of violacein production by the test strains. Violacein production was measured spectrophotometrically (refer to Materials and Methods). Data are shown as the mean ± SD of absorbance at 585 nm. The * and ** represent statistical differences from controls at the *P* < 0.05 and *P* < 0.01 level respectively. C. Biofilm dispersal activity (crystal violet assay) of extracts from the isolated strain *V. alginolyticus* 12. Bacterial extracts at different concentrations (1, 5 and 10 μg ml^−1^, w/v) were tested against a biofilm‐forming reference strain, *P. aeruginosa* PAO1. Tests performed with furanone and methanol only (solvent carrier) acted as the positive and negative controls respectively. Different letters (a or b) indicate a statistically significant difference between experimental groups and the control (*P. aeruginosa* culture). D. Effects of the QSI compound on *P. aeruginosa* PAO1 growth in LB media, with (yellow line) and without (blue line) the 12^#^ strain extract (10 μg ml^−1^). The extract did not affect the bacterial growth rates. Data are shown as means ± SD (*n* = 3).

None of the tested strains (*V. alginolyticus*,* L. fusiform*,* S. hominis*,* B. cereus*, and *S. warneri*) had any apparent effect on the colony‐forming units of *C. violaceum* 12472 (Fig. 2A). However, we observed that these five bacterial species significantly lowered violacein content, especially *V. alginolyticus*12 (which reduced violacein production by 95.7%, *P* < 0.01) (Fig. 2B). Therefore, the reduced production of violacein by bacterial culture was not due to the reduction of the ‘quorum’, but rather because ‘sensing’ was somehow interrupted.

### Extract *V. alginolyticus* 12 inhibited the formation of biofilm

For inhibitory ability, the extract (*V. alginolyticus* 12) at 1 and 5 μg ml^−1^ had little effect on the existing biomass of *P. aeruginosa* PAO1 biofilms after treatment for 24 h (Fig. S1A). At 10 μg ml ^−1^, the extract exhibited a significant direct biofilm reduction effect, although bacterial counts were not affected (Fig. S1B), perhaps because the H12 extract did not exert direct bactericidal effects on pre‐existing *P. aeruginosa* in the biofilm. Subsequently, the antibiofilm activity of the potent extract (*V. alginolyticus* 12) was tested against the wild‐type of the widely used biofilm forming isolate *P. aeruginosa* PAO1. Figure 2C shows the quantitative analysis of PAO1 biofilm inhibition. A dose‐dependent pattern of inhibition was observed with PAO1 biofilm in response to *V. alginolyticus* 12 extract treatment. The most significant effect appeared at a concentration of 10 μg ml^−1^, which caused the biofilm formation to decrease by 37.5% (*P* < 0.05). Visualization attachment analysis by light microscopy also revealed a considerable reduction in biofilm intensity (Fig. S2). Interestingly, we found no statistically significant effect upon the growth of *P. aeruginosa* in the presence of bacterial extracts (Fig. 2D).

Confocal laser scanning microscopy (CLSM) *z*‐stack three‐dimensional images provided us with a precise evaluation of the structure of the *P. aeruginosa* PAO1 biofilm at different time points (12 and 36 h). This revealed reductions in the total biomass and thickness of the biofilm in the group treated with H12 strain extract. Considering its topology, a well‐grown biofilm with adherent bacterial cells was observed in the negative controls (i.e. a normal biofilm developed by *P. aeruginosa* PAO1), whereas dispersed bacterial cells were observed in the positive control (Fig. S3, b1 and b2) and the treated sample (Fig. S3, a1 and c1). Extremely thick biofilms (containing more cells and polysaccharides) were formed in the negative control relative to those of the experimental group. Furthermore, images clearly showed a disrupted surface topology and height distribution profile of the biofilm that developed in the presence of the *V. alginolyticus* 12 extract when compared with the control biofilm (taking 36 h as an example; Fig. S3, a2 and c2). In the negative control group, *P. aeruginosa* PAO1 developed a thick, dense biofilm after 12 h, whereas on a surface coated with the *V. alginolyticus* 12 bioactive crude extract, biofilm formation and bacterial adherence were prevented (Fig. S3, a1 and c1). At 36 h, corresponding to the mature period of biofilm, viable *P. aeruginosa* cells accounted for a larger proportion than found in the negative control, which confirmed that strain H12 extract was not toxic to cells within the biofilm. Furthermore, the large proportion of live cells also demonstrated that the QSI extract delayed the biofilm's development (Fig. S3, a2 and c2).

### Extract *V. alginolyticus* 12 reduced the production of QS‐regulated virulence factors

**Figure 3:**
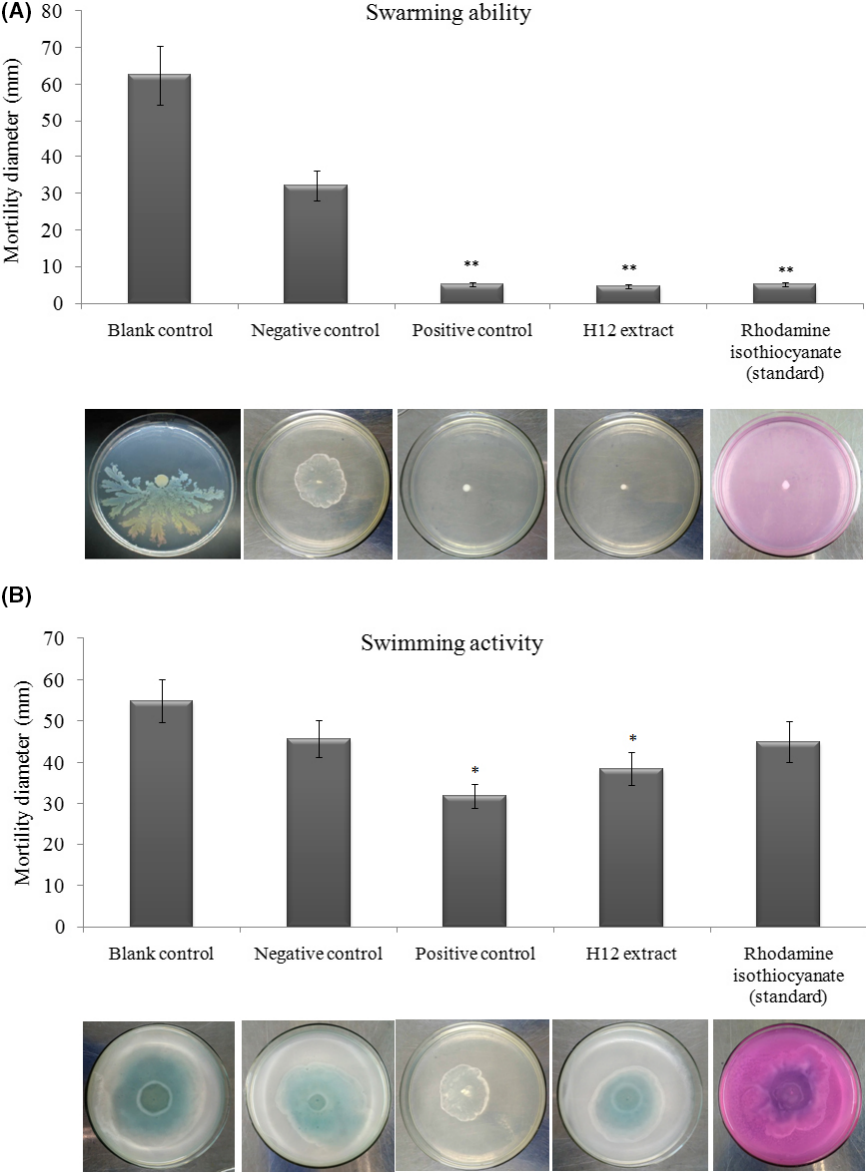
Effects of H12 extract on *P. aeruginosa* PAO1 motility. A. swarming ability and (B) swimming ability. The assay was conducted on plates containing 0.3% agar in the absence of H12 extract (10 μg ml^−1^). Blank control (ddH_2_0), negative control (methanol only), positive control (furanone) and rhodamine isothiocyanate (standard) were also assayed at the same time. Swarming and swimming motility diameters were measured using a caliper. The data represent mean values from three independent experiments performed in duplicate. Values represent means ± standard deviation. The * and ** are statistically different from the negative control at the *P* < 0.05 and *P* < 0.01 level respectively.

The effects of H12 extract on the swarming motility of *P. aeruginosa* were determined by inoculating overnight cultures of *P. aeruginosa* PAO1 onto motility plates; results are shown in Fig. 3A. *P. aeruginosa* PAO1 exhibited swarming motility on soft agar plates (blank control and negative control) at the point of inoculation with a swarming diameter between 32.4 and 62.5 mm. In the presence of H12 extract (10 μg ml^−1^) (test group), the results were similar to the positive control (furanone) and the rhodamine isothiocyanate standard in that the swarming ability was significantly reduced (*P* < 0.01) – only a small colony was formed in the centre with a diameter of approximately 5 mm. In addition, we failed to observe tendril formation, or other features indicative of swarming motility.

For the swimming motility (Fig. 3B), in the absence of H12 extract (10 μg ml^−1^), the swimming motility zone for *P. aeruginosa* PAO1 was 40.1 ± 6.6 mm. This diameter was lower (*P* < 0.05) than that observed for the blank control in its normal state (55.9 ± 7.4 mm for swimming). The presence of furanone (the positive control) resulted in a significant reduction (*P* < 0.05) in swimming motility to 31.9 ± 4.2 mm. However, the rhodamine isothiocyanate standard did not inhibit the swimming ability of *P. aeruginosa* PAO1 compared with the control; at best, it exerted a weak inhibitory effect that was not statistically significant (*P* > 0.05) (Fig. 3B).

**Figure 4:**
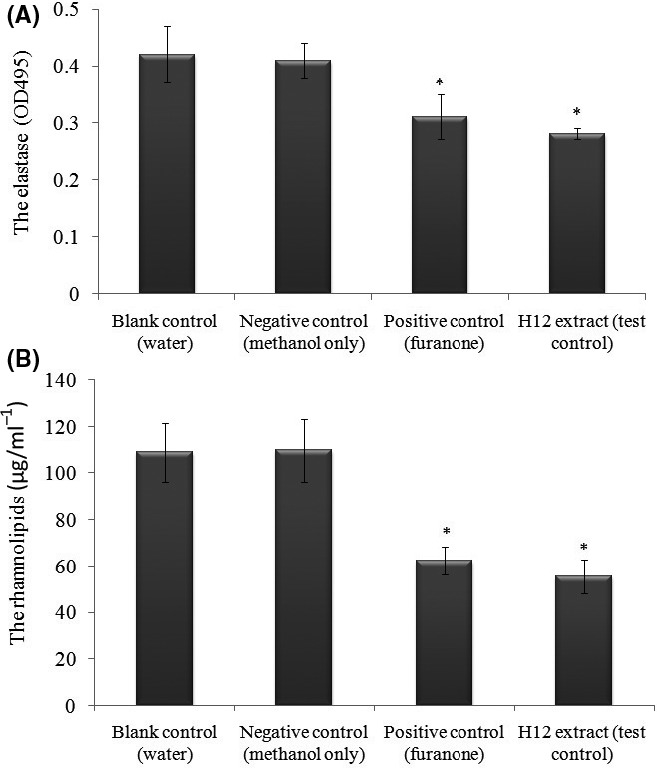
Effect of H12 extract on elastase activity (A) and rhamnolipid product (B) in *Pseudomonas aeruginosa* PAO1. Error bars indicate the standard deviations of three measurements. *P < 0.05 compared with the negative control group.

Another two forms of QS‐related virulence factors, elastase and rhamnolipid, were also analysed spectrophotometrically to assess the effects of H12 extract on the QS system of *P. aeruginosa*. As shown in Fig. 4, the production of the two QS‐relevant virulence factors was significantly reduced by 10 μg ml^−1^ of H12 extract. H12 extract clearly reduced elastase activity in the supernatant of treated PAO1, compared with the untreated PAO1 supernatant (blank control and negative control) (*P* < 0.05) (Fig. 4A). Similarly, the production of rhamnolipid by PAO1 cells was also inhibited by 45.5% in the presence of target extract (Fig. 4B).

### Identification of QSI compounds

**Figure 5:**
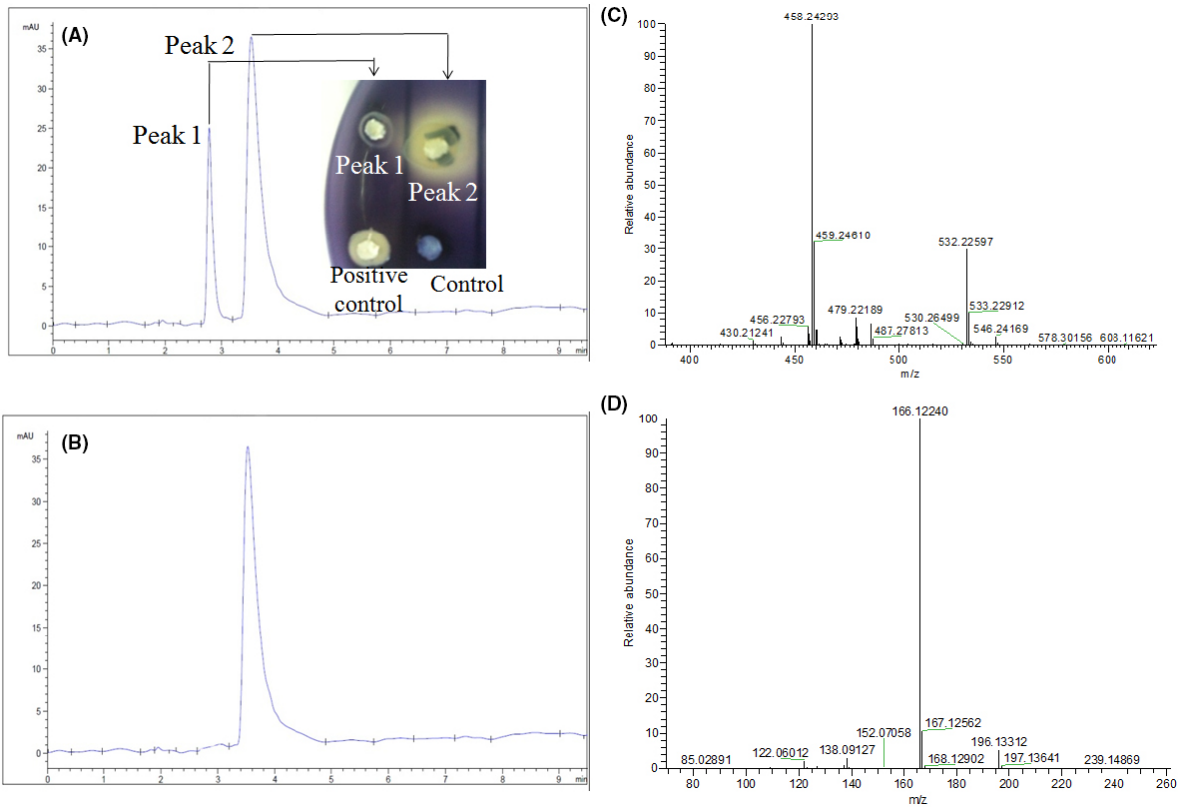
Analysis of bioactive fractions showing quorum‐sensing inhibition. Pre‐HPLC (A) and UPLC (B) analysis of the *V. lyticus* 12 extract. The chromatogram shows its two main active fractions. The inset image in (A) is the re‐test of QSI activity of the two peak compounds. In the inset image, the “60% methanol + collected extract” and “60% methanol only” represented the positive and negative controls respectively. The GC/MS chromatograms (positive ions) (C) and GC/MS chromatograms (negative ions) (D) of the active fraction (peak 2) of the *V. alginolyticus*12 extract. Peaks represent a function of intensity measured in milli‐absorption units over time (min).

**Figure 6:**
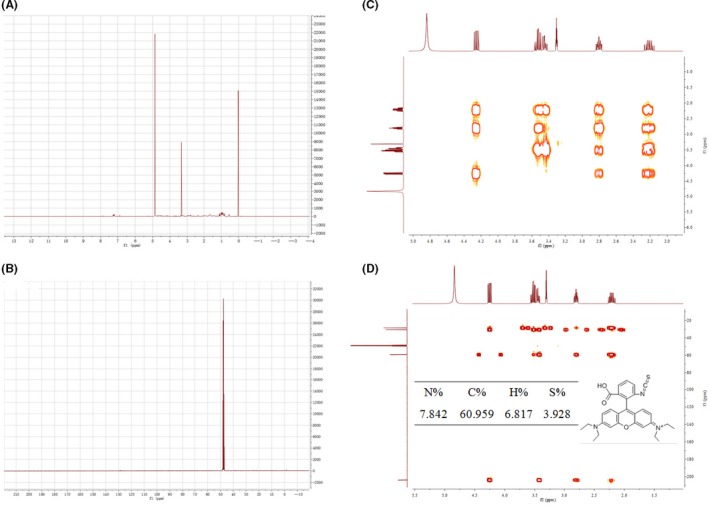
Nuclear magnetic resonance spectroscopy analysis of H12 extract. A. ^1^H‐NMR spectrum of compounds in methanol‐d4 at 400 MHz. B. ^13^C‐NMR spectrum of compounds in methanol‐d4 at 100 MHz. C. and (D) show the 2D‐HMQC and 2D‐HMBC chromatograms respectively. The inset picture in (D) shows the elemental composition and possible chemical structure of rhodamine isothiocyanate (re‐drawn using ChemBioDraw Ultra v12.0).

After separating crude extracts from the pre‐HPLC, two main peaks were collected and re‐detected individually for their QSI activity (Fig. 5A). Fraction peak 2, collected via the C18 cartridge with 60% methanol, showed a bioactivity zone of inhibition; consequently, it was selected for further characterization by ultra‐performance liquid chromatography (UPLC) analysis: the chromatogram showed a single peak at a retention time of 3.54 min (Fig. 5B). A mass spectral peak, detected at positive m/z 458.242 and negative m/z 166.122, was considered to be the corresponding experimental mass of the active fraction (Fig. 5, C&D). Combining the GC‐MS analysis with nuclear magnetic resonance (NMR) (C^13^ and H^1^) (Fig. 6, A and B), the calculated (or theoretical) molecular mass of the compound was 500.6. The molecular mass of the active fraction was further confirmed by two‐dimensional heteronuclear multiple quantum correlation (HMQC) and heteronuclear multiple bond correlation (HMBC) (Fig. 6, C and D). The elemental proportions of C, N, H and S were 60.959%, 7.842%, 6.817% and 3.928%, respectively and detected spectra resembled rhodamine isothiocyanate (C_29_H_30_N_3_O_3_S). The identified target product was tentatively referred to as rhodamine isothiocyanate analogue.

**Figure 7:**
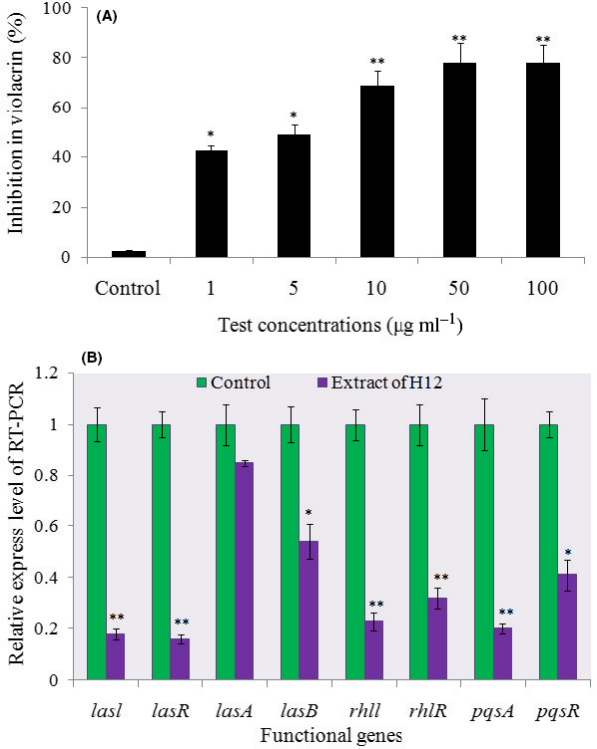
A. The violacein Inhibitory activity analysis. The proportion (%) of violacein inhibition in response to pure rhodamine isothiocyanate applied at six different concentrations (0, 1, 5, 10, 50 and 100 μg ml^−1^). Data shown are the mean ± SD of three replicates. The * and ** denote statistical differences from the control at the *P* < 0.05 and *P* < 0.01 level respectively. B. Expression profiling of some QSI regulatory genes from *P. aeruginosa* under *V. alginolyticus* 12 extract (10 μg ml^−1^), as measured by qRT‐PCR. The results are based on three independent experiments. The mRNA expression of these genes in the absence of the extract served as a control. Data are presented as means ± SD (*n* = 3). *P < 0.05 or **P < 0.01 in comparison with the control.

To confirm that the QS inhibitory activity produced by strain *V. alginolyticus* 12 was elicited by the target compound, rhodamine isothiocyanate standard was purchased from Sigma (Sigma, St. Louis, MO, USA, CAS No. 36877‐69‐7). This commercial product's QSI activity was tested using the same methodology described in Experimental Procedures section (determination of growth and violacein production). As Fig. 7A shows, a concentration‐dependent inhibitory activity was observed, and all of the tested concentrations (1, 5, 10, 50 and 100 μg ml^−1^) of rhodamine isothiocyanate significantly inhibited the violacein content (range of reduction: 43.7–78.1%).

### Expression analysis by qRT‐PCR

A near 1.82‐ to 6.23‐fold downregulation of the genes responsible for base‐AHL (acyl‐homoserine lactone) biofilm formation (*lasB*,* lasI, lasR, pqsA* and *pqsR*) was observed in the *P. aeruginosa* strain (*P* < 0.05 or *P* < 0.01) (Fig. 7B). The expression of two virulence‐related genes, *rhlI* and *rhlR*, was also significantly reduced, by 75.4% and 68.2% respectively (*P* < 0.01). The expression of the *lasA* gene in PAO1 was slight decrease and was not significantly different when compared between experimental and control groups (Fig. 7B).

## Discussion

Biofouling is a serious global problem in marine environments, causing extensive material and economic costs worldwide. Early antifouling strategies relied on physical cleaning, or the use of antibacterial compounds, detergents, aldehydes, oxidizing agents or diverse biocides such as tributyltin. All of these compounds, especially when used in large amounts, or in confined environments, pose serious health and environmental problems due to their toxicity or ecotoxicity (Voulvoulis *et al*., 2002; Yebra *et al*., 2004; Thomas and Brooks, 2010). The search for QSI compounds has since turned to the ocean and its abundant variety of organisms, many of which are already known to produce useful bioactive compounds, such as halogenated furanones produced by the red alga *D. pulchra* (Rasmussen *et al*., 2000), brominated alkaloids from *Flustra foliacea* (Peters *et al*., 2003), and kojic acid from *E. knighti* (Dobretsov *et al*., 2011). More recently, the culture supernatant from Antarctic marine bacterium *P. haloplanktis* TAC125 was shown to impair the formation of *S. epidermidis* biofilm (Papa *et al*., 2013; Parrilli *et al*., 2015). These findings demonstrate that QSI compounds are valuable antifouling agents in that they could serve as potential effective additives to environmentally friendly commercial antifouling paints due to their non‐toxicity (Tello *et al*., 2012).

The biofouling process begins with bacteria that permanently attach themselves and form complex biofilms. Because this bacterial biofilm formation is, in part, controlled by QS, various QSI strategies have been harnessed from marine microorganisms as compounds which could be used to interfere with bacterial QS, and thus represent novel forms of antifouling agents (Qian and Dahms, 2008; Dobretsov *et al*., 2011). Among the array of marine niches, screening for antifouling compounds from coral symbiotic microbes is of particular interest because of their high biodiversity and rich secondary metabolism (Mohamed *et al*., 2008; Fusetani, 2011; Karina *et al*., 2013). In our present study, we found that 15% of coral‐associated bacteria exhibited inhibition activity against *C. violaceum* 12472. Previous studies reported that approximately 11–24% of the coral‐associated bacteria sampled were capable of QS inhibition activity against *E. coli* pSB1075, *C. violaceum* CV026 and *A. tumefaciens* KYC55 indicator strains (Romero *et al*., 2011; Karina *et al*., 2013). Our results are in line with these proportions and further suggest that coral ecosystems represent an ideal reservoir from which to develop QSI products.

Previous studies also suggested that coral‐associated *Vibrio* spp. can interfere with QS and subsequently inhibit the formation of biofilms (Tait *et al*., 2010; Alagely *et al*., 2011). Indeed, in the present study, strain H12 stood out in particular and the presence of 16s rRNA confirmed that this strain belonged to the *Vibrio* genus (hence, we named this strain *V. alginolyticus* 12). Using fermentation broth extract from this particular strain, we observed a 37.5% inhibition of the biofilm's biomass at the highest concentration of H12 extract (Fig. 2C). This result indicates that bioactivity exudates were potentially present in the extracellular matrix, and that QSI activity compounds are capable of interfering with the QS system to influence the formation of biofilm in the model G^−^ strain PAO1. Interestingly, *V. alginolyticus* 12 did not affect the growth of *P. aeruginosa* (Fig. 2D). Similarly, when the cytotoxic effects of isothiocyanate on the mammalian cell line L929 were investigated, they also showed no cytotoxic effects (Borges *et al*., 2014). These characteristics suggest that the target compounds are relatively safe antifouling agents, which should promote their broad‐scale development and application as QSI compounds.

HPLC and GC‐MS identified rhodamine isothiocyanate (C_29_H_30_N_3_O_3_S) analogue as the bioactive fraction (Fig. 6D). When this compound was applied as a treatment, it caused a significant reduction in biofilm density and thickness in the form of a flattened biofilm lacking structural heterogeneity, whereas *P. aeruginosa* formed a highly structured biofilm in the absence of this compound. Z‐stack images from confocal laser scanning microscopy showed this reduction clearly (Fig. S3). Light microscopy also revealed a considerable effect upon the mean thickness and roughness coefficient in the treatment group (data not shown). To the best of our knowledge, this is the first report of rhodamine isothiocyanate, or its analogue, functioning as an antibiofilm agent from marine *Vibrio* spp. From the analysis of chemical structure, it is evident that the hexatomic ring appears in several types of AHL signal, such as HHQ and PQS (Grandclement *et al*., 2016). The inhibition properties of this agent may arise from competitive binding with the transport protein that interrupts the transcription of the AHL signal. Several types of isothiocyanate‐benzylisothiocyanate (BITC), 2‐phenylethylisothiocyanate (PEITC) have been reported to exhibit QSI activity and are also known to affect the QS system, based on the high structural similarity among signal molecules (Borges *et al*., 2014). This result supported our hypothesis that AHL‐based analogues can operate as QS modulators or antibiofilm agents. Because the QSI activity was confirmed when pure commercial rhodamine isothiocyanate was used, it led us to propose that the QS inhibitory activity produced by strain H12 is driven by rhodamine isothiocyanate or its analogue.

We should highlight that most of the known antibiofilm molecules also exhibit antibacterial activity (bactericidal or bacteriostatic). Some studies have demonstrated that both QSI activity and antimicrobial activity may co‐occur (Busetti *et al*., 2014). Our results indicated that the absence of violacein was mainly caused by QS disruption (Fig. 2B), especially since the presence of rhodamine isothiocyanate reduced the biofilm biomass without affecting the viability of PAO1 cells living in the biofilm (Fig. S3). This would suggest that the target compound affects adhesive properties of the biofilm without affecting its bacterial viability. This property makes rhodamine isothiocyanate particularly interesting to couple with other conventional antifouling agents in a synergistic framework.

To further understand the QSI and antibiofilm potential of rhodamine isothiocyanate at the molecular level, and to lend support to the outcome of our *in vitro* results, we followed up with qRT‐PCR analysis. We know that QS genes play a crucial role in the synthesis of EPS, the most critical component of biofilm, and in biofilm maturation. In particular, *lasI* regulates the expression of several genes involved in the production of the glucose rich EPS‐matrix. In the PAO1 system, *lasI* and *rhlI* are involved in autoinducer synthesis, while *lasR* and *rhlR* code for transcriptional activators (Sharma *et al*., 2014). In our study, the expression of *lasB*,* lasI*,* lasR*,* rhlI* and *rhlR* was reduced considerably (Fig. 7B), thus indicating that the *V. alginolyticus* 12 extract is able to inhibit both the *las‐* and *rhl‐*regulatory systems. This also points to a plausible mechanism for its QSI activity, perhaps through an interaction with the *las‐* and *rhl‐* donor or receptor. Work by Vattem *et al*. (2007) achieved a similar result to ours with *Kigelia africana* extracts. In addition to its antibiofilm activity, our extract also inhibited the production of *P. aeruginosa* AI‐2 factors, such as the *pqs* genes (Fig. 7B). Our prior work had revealed the inhibition of elastase activity and siderophore production by *Rhizobium* spp. NAO1 occurs via interference with QS activity, because these virulence factors are under the control of the *lasI‐lasR* and *rhlI‐rhlR* systems (Chang *et al*., 2017). Overall, these studies suggest that the potential target (rhodamine isothiocyanate) exhibits QSI activity against *P. aeruginosa* PAO 1 via a significant reduction in the QS cascades via downregulated AI‐1 or AI‐2 genes.

**Figure 8:**
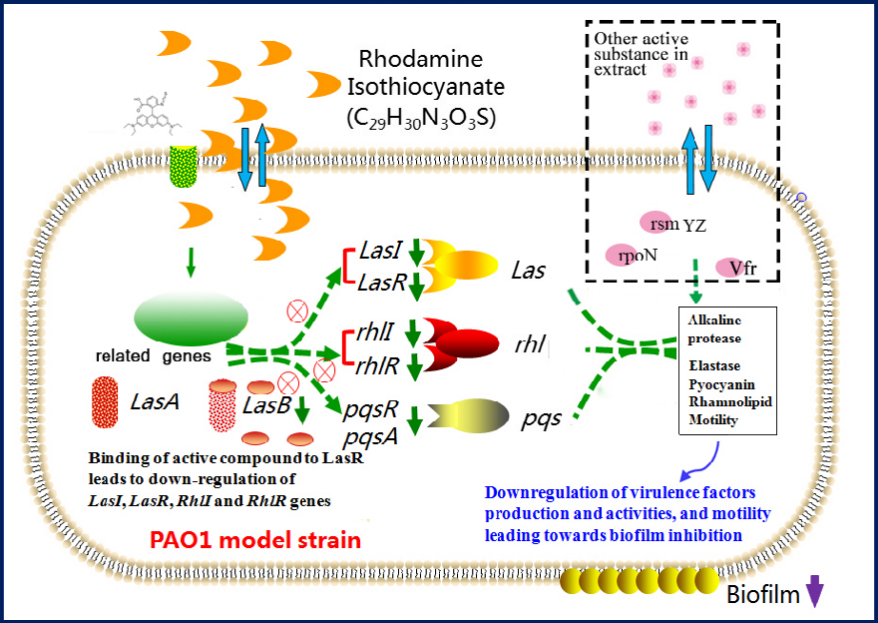
A hypothesized model to explain the possible mechanism (transcriptional regulation) of QSI activity in *P. aeruginosa* PAO1.

The common mechanisms underlying QS interference include the inhibition of signal biosynthesis, enzymatic signal degradation, and disruption of reception signal molecules (Song *et al*., 2018). Based on our results, we propose a model, shown in Fig. 8, to explain the transcriptional regulation of QSI in *P. aeruginosa*. It is possible to infer that the QSI effect in the *C. violaceum* 12472 systems, with rhodamine isothiocyanate or its analogue, may be mediated by interference with the synthesis or activity of AHL molecules. This model indicates that the target compound would disrupt the *las* and *rhl* pathways, there by influencing protein activities downstream (such as the function of protease, elastase, pyocyanin and rhamnolipid), eventually reducing the production of biofilm in combination with the *pqs* pathway. Indeed, we saw a reduction in elastase activity and rhamnolipid production (Fig. 4), thus providing supporting evidence for our model. Therefore, QSI activity can arise from a combination of two mechanisms: interference with the activity of CV12472 to produce AHLs by the *LuxR* system and modulation of AHL synthesis by the *LuxI* system. In addition, *V. alginolyticus* 12 has the potential to reduce the virulence of *P. aeruginosa* by interfering with the appropriate production of AI‐2 factors (such as *pqs* genes).

It should be noted that the swimming ability of PAO 1 can be reduced by H12 extract (Fig. 3B), which is similar with Deziel *et al*. (2001) results. However, Beatson *et al*. (2002) pointed out that swimming motility in *P. aeruginosa* was not regulated by QS molecular. Based on this, we speculated that besides QS signals, the target compound (or other activity substance in the extract) probably has a globally role to regulate biofilm formation factors in PAO 1, such as *vfr*,* rpoN*, small RNAs (*rsmYZ*) and cyclic diguanylate monophosphate (c‐di‐GMP) regulator (Yuan *et al*., 2015) (the dotted circle in Fig. 8). Further studies to confirm this hypothesis would be carried out in the future work.

In summary, this study identified QSI activity by a *Vibrio* species isolated from coral; the putative compound responsible for this effect was identified as rhodamine isothiocyanate. The analogue molecule(s) of rhodamine isothiocyanate may have disrupted the AI system in *P. aeruginosa*; confocal microscopy confirmed that the *P. aeruginosa* biofilm featured a reduced thickness and biomass. Our qRT‐PCR analysis suggested an underlying molecular mechanism that affected functional genes involved in the formation of biofilms and virulence production. Interestingly, the extract did not reduce the growth of *P. aeruginosa*. These findings may facilitate the discovery of novel antifouling agents with greater efficacy against biofouling. Further studies are now underway in our laboratory to develop a cartridge delivery system.

## Experimental procedures

### Isolation of coral symbiotic bacteria

Coral samples (*P. damicornis*) were collected from Xishan Island (3°57.058′E, 36°8.532′S) in the South China Sea. Samples were collected from six sites at a depth of 5–6 m. The salinity was approximately 33.1‰ and the seawater temperature was 29.7°C. At each site, five coral samples were collected. These were washed with sterile seawater, homogenized by grinding and agitation, and serially diluted in sterile seawater. Next, 50 μl dilutions (of 10^−4^ to 10^−7^) were surface‐plated onto marine agar 2216 (BD Difco, Franklin Lakes, NJ, USA) and incubated at 30°C for 48–72 h. A quantity of pre‐test colonies, chosen on the basis of their differential colonial morphology, were collected using sterile toothpicks and incubated for 8 h at 30°C in Luria broth (LB: consisting of 10 g l^−1^ typtone, 5 g l^−1^ yeast extract, 10 g l^−1^ NaCl, 1 5 g l^−1^ agar, sterilized at 121°C).

### Bioreporter strains and growth conditions

The *C. violaceum* ATCC 12472‐reporter strain was used to screen QSI potential bacteria. We chose this strain because it can regulate pigment production via QS signal and is readily inhibited by QS inhibitors. Samples were visually screened for their QS inhibition activity by observing the ensuing violacein production (McLean *et al*., 2014). *Pseudomonas aeruginosa* PAO1 is an established model species that was used throughout this study to prepare biofilms. Both strains were cultured in LB medium, either in liquid form or solidified form, using 1.5% agar as needed.

### Screening of the QSI potential bacteria

A modified 24‐well plate assay method was carried out with the *C. violaceum* ATCC 12472 strain to detect any QSI activity. In brief, a 0.22 μm filter film was established following the soft‐agar technique (Teasdale *et al*., 2009); due to its presence, the test bacteria and reporter strain were separated to avoid physical contact. Small‐molecule compounds, secreted by the test strains, could penetrate the filter film and “sense” the reporter strain. The screened isolates were cultured overnight in LB medium at 30°C in 1.5 ml microcentrifuge tubes with constant shaking at 180 rpm. Then, 100 μl of bacterial suspension was added to 900 μl of soft LB medium and cultured in a 24‐well plate. A 0.22 μm filter film was gently placed on the surface of the media. The upper filter film layer inoculated with *C. violaceum* ATCC 12472 (OD_600_ near 0.1) was used to screen the potential QSI strains. The *C. violaceum* ATCC 12472 bacterial suspension (10 μl) was pipetted onto the filter paper, and bacteria were incubated in quarantined polystyrene (24‐well) plates. In this plate‐based bioassay, we used 2,5‐Dimethyl‐4‐hydroxy‐3[2H]‐furanone (Sigma‐Aldrich, St. Louis, MO, USA, CAS No. 3658‐77‐3) and LB broth to act as positive and negative controls respectively. After incubation for 24 h at 30°C, the inhibition of pigment production on the filter paper was assessed. Positive QSI activity was recorded as visible colourless paper. The bacterial isolates that showed promising positive QSI activities were then selected for further study. To ensure the reliability of this experiment, the QSI activities of the selected isolates were independently tested three times.

### Identification of bacterial strains

The potential QSI strains were grown overnight in LB broth at 30°C; then 200 μl from each culture was transferred into a clean 1.5‐ml microcentrifuge tube and centrifuged at 8000 rpm for 1 min. Flow‐through was then discarded, 100 μl of TE buffer was added, and the sample was gently mixed, then boiled for 10 min. The ensuing supernatant contained the DNA crude extract (OD_260_/OD_230_ > 1.7; OD_260_/OD_280_ between 1.8 and 2.0). The 16S rRNA gene (~1500 bp) was then amplified using the forward primer 27F (5′‐AGAGTTTGATCCTGGCTCAG‐3′) and the reverse primer 1492R (5′‐GGTTACCTTGTTACGACTT‐3′) (Weisburg *et al*., 1991), and then sequenced at BGI‐Shenzhen (BGI, Shenzhen, Guang dong, China). The sequences obtained were assembled, analysed, and manually edited with a CAP3 software package. The resulting sequences were compared against those from the NCBI database (http://www.ncbi.nlm.nih.gov) in a BLAST analysis.

### Determination of growth and violacein production

To rule out the possibility that an antimicrobial effect was responsible for inhibiting purple pigment production, a growth experiment, using different test strains, was conducted. Effects upon growth were determined via the colony‐counting plate method (Choo *et al*., 2006). The *C. violaceum* ATCC 12472 was serially diluted and 100 μl of diluted cultures was spread onto LB plates, which were incubated at 30°C for 24 h. Bacterial counts were then compared with the control.

To quantify violacein production, a 1 ml culture was centrifuged at 12000 rpm for 10 min to precipitate any insoluble violacein. The culture's supernatant was then discarded and 1 ml of methanol was added to the pellet. The solution was vortexed vigorously for 30 s to completely solubilize the violacein, then centrifuged at 12000 rpm for 10 min to remove the cells (Choo *et al*., 2006). Next, 200 μl of the violacein‐containing supernatant was added to 96‐well flat‐bottomed microplates, with three wells used per solution; absorbance was read with a spectrophotometer (Infinite^®^ 200 PRO, Tecan, Groedig, Salzburg, Austria) at a wavelength of 585 nm (Blosser and Gray, 2000).

### Inhibiting the biofilm of PAO1

The effect of an QSI positive strain on the biomass of biofilms produced by *P. aeruginosa* PAO1 was determined using the crystal violet (CV) method (Choo *et al*., 2006). In brief, fresh cultures of PAO1 were added to 96‐well polystyrene plates (100 μl per well) and incubated in LB medium (Hinsa and O'Toole, 2006), to which bacterial extracts (1, 5 and 10 μg ml^−1^, w/v) were then added. The mixtures were incubated at 30°C for 48 h. Planktonic cells and the spent medium were then removed from each culture; the remaining adherent cells were gently rinsed twice using deionized water. To each well, a 100 μl CV solution (1%, w/v) was added and left for 30 min at room temperature. The excess dye was discarded, and the plates were washed gently using deionized water. The CV‐stained cells were then solubilized in methanol and their absorbance at 600 nm determined by a microplate reader (Infinite^®^ 200 PRO; Tecan). *P. aeruginosa* PAO1 cultures incubated without the target strain extract served as negative controls and purified water was used as a blank‐control. Experiments were performed with 12 replicates (12 replicate wells in the 96‐well plates) per treatment. When absorbance was determined, three readings were recorded for each well.

We then examined the biofilm‐reduction effects and biofilm structure under H12 extract treatment. Biofilm dispersion was assessed according to methodology described by Luo *et al*. (2017). Pre‐sterilized glass microscope slides were used to observe the structure of biofilms under CLSM, as described by Tolker (2014). In brief, cells were grown in LB medium overnight and diluted with fresh medium to an OD_600_ of approximately 0.02. Then, 4 ml dilutions were incubated with (10 μg ml^−1^, w/v) or without the QSI extracts in 6‐well plates, with a glass microscope slide in each well, under static conditions. After 24 h, the glass slides were withdrawn and rinsed with deionized water to remove loosely attached cells. The biofilms on one side were stained with 5 μM SYTO9 and 5 μM PI dye (Sigma) in the dark, while those on the other side were simply wiped off. After 15 min, the slides were washed, and observed under CLSM (LSM 710; Zeiss, Jena, Thuringia, Germany) with a 60× objective lens to visualize the biofilms. The 488‐nm excitation and 520‐nm emission filter settings were used to detect SYTO 9, while the 488‐nm excitation and 620‐nm emission filter settings were used for PI. We also quantified biofilm parameters from the CLSM images using COMSAT software (Heydorn *et al*., 2000). The total biomass, mean thickness, and roughness coefficient of biofilms were also evaluated. To produce 3D transmission‐fluorescence photos of *P. aeruginosa* PAO1 biofilms, we used FV10‐ASW2.0 Viewer (Olympus, ?Shinjuku, Tokyo, Japan). Optical sections were 5 μm apart on the Z‐axis and images were acquired at 640 × 640 pixels with a 12‐bit intensity resolution. Digital images were processed in Leica Confocal Software Lite (Leica Microsystems, Germany).

### Growth curve assay

To determine the effects of an extract upon the growth of *P. aeruginosa* PAO1, a growth curve assay was conducted following Chang *et al*. (2017). *P. aeruginosa* PAO1 was cultured in LB broth with and without the strain H12 extract (10 μg ml^−1^, w/v). Cultures were incubated at 30°C; every 0, 3, 6, 9, 12, 18, 27, 36 and 48 h, the optical density at 600 nm was determined using a microplate spectrophotometer (Infinite^®^ 200 PRO; Tecan).

### Swarming and swimming motility assay

Five millilitres of molten soft top agar (0.3 g agar, 1.0 g tryptone, 0.5 g Yeast extract powder, 0.5 g sodium chloride, 100 ml deionized water) was prepared containing H12 extract at a final concentration of 10 μg ml^−1^. Then, this agar was poured immediately over the surface of a solidified LB agar plate as an overlay and allowed to dry for 3 h at 30°C. The plates were then centre point inoculated with PAO1 (1 × 10^7^ CFU ml^−1^) and incubated at 37°C for 24 h. The extent of swarming was then determined by measuring the diameter of the motility swarms (Rashid and Kornberg, 2000).

*Pseudomonas aeruginosa* PAO1 swimming motility was measured according to Wu *et al*. (2011). Briefly, diluted *P. aeruginosa* PAO1 at 1 × 10^7^ CFU ml^−1^ was point inoculated with a sterile toothpick onto plates containing 0.3% (w/v) soft agar (as mentioned above) in the presence or absence of H12 extract. The plates were then incubated for 24 h at 37°C. The diameter of the migration distance around the incubation site was also measured.

### The elastase and rhamnolipid assay

Elastase activity was measured by using previously described methodology, with some modification (Ohman *et al*., 1980). In brief, 100 ml of culture supernatant was mixed with 900 μl of elastin‐congo red (ECR) buffer (100 mM Tris, 1 mM CaCl_2_, pH 7.5) containing 20 mg ECR (Sigma Chemical Co., St. Louis, MO, USA). This mixture was then incubated at 37°C for 3 h in a water bath. The samples were then centrifuged (1500 rpm for 10 min at 4°C) to remove insoluble ECR. The absorbance of the supernatant from both control and treated samples was determined by reading OD_495_. The proportional change (%) in absorbance was then calculated to determine the reduction in elastase activity.

The production of rhamnolipid in *P. aeruginosa* PAO1 was then assayed as previously described (Boles *et al*., 2005; O'Toole, 2011). *P. aeruginosa* biofilms were then assayed in a 96‐well polystyrene microtitre plate. After cell viability was assayed by measuring the OD at 600 nm, extracted rhamnolipid was measured at 420 nm, and biofilm cells attached to the well surface were assayed using crystal violet staining.

### Extracting QSI active components

The potential positive strain (H12) was incubated for 48 h in LB broth at 30°C while shaken at 180 rpm. A 1 l bacterial culture was centrifuged for 15 min at 12000 rpm, and its supernatant collected. This cell‐free supernatant was then mixed with the same volume of ethyl acetate (EtOAc) to separate the organic phase. Repeated shaking ensured the soluble fraction was completely separated. Ethyl acetate extract was then dry‐evaporated under vacuum at 45°C in a rotary evaporator. Organic residues were then dissolved in methanol (0.5 ml) for subsequent use. The methanol‐dissolved extract was then filtered through a 0.22‐μm filter film to completely remove any impurities.

The QSI activity of the prepared extracts was then re‐verified using a standard disc diffusion assay (Busetti *et al*., 2014). In brief, an overnight culture of the reporter strain CV12472 (100 μl) was poured into soft‐layer LB media (5 ml) and then plated and allowed to solidify. The putative extracts – from the H12 strain identified in the preliminary screen – were then pipetted onto filter paper, and the appearance of turbidhalo pigmentless areas of the CV12472 violet colour was designated as positive QSI activity. The positive extracts were stored at −20°C and used in the experiments described below.

### Chromatographic analysis of active compounds

Extracted compounds were separated via preparative high performance liquid chromatography (pre‐HPLC) processing. Extract samples (100 μl) were injected into a reverse phase C18 core‐shell column (50 × 2.1 mm, Waters, USA) through an autosampler (ThermoFisher Scientific, Waltham, MA, USA). The mobile phase used 83% methyl alcohol and 17% water at a flow rate of 0.5 ml min^−1^ at 30°C. The fraction collector gathered the samples, separated by 30s in a series. As the separation process ended, every peak sample was obtained and concentrated in a rotary evaporator. Finally, each purified sample was re‐tested to confirm it exhibited QSI activity (as described earlier).

The collected QSI peaks were further purified on a reversed‐phase UPLC system (Waters Delta Prep 4000; Mckinley Scientific, Sparta, NJ, USA) using a C18 column and a linear water/acetonitrile gradient that contained 0.1% trifluoracetic acid. The purity of the fraction was tested by analytical UPLC (Agilent 1100; Agilent, Santa Clara, CA, USA) with the same gradient. The fraction collected was lyophilized, and the residue dissolved in 1 ml of acetonitrile/water (1:1, v/v) to determine its molecular weight by MS on a LTQ XL Orbitrap using a static nanospray (Thermo‐Fisher, San Jose, CA, USA) in the positive/negative ions mode. To determine the molecular structure of bioactive compounds, a purified and active QSI sample was subjected to NMR spectroscopy to obtain its 2D heteronuclear single quantum coherence and HMBC spectrogram.

### Influence upon the expression levels of QS regulatory genes

*Pseudomonas aeruginosa* PAO1 was grown in 10 ml of LB liquid medium until its OD_600_ was almost 0.1. At this time, approximately 10 μl of extract (concentration: 10 μg ml^−1^) of strain H12 was added to the PAO1 culture medium to form the treatment groups. The extract was dissolved in methanol [to a final concentration of 0.1% (v/v) methanol]. The group receiving only 10 μl of methanol acted as the solvent control. After 24–36 h, total RNAs were extracted from the control and treated *P. aeruginosa* with TRIzol (Takara, Dalian, China) and reverse transcribed into cDNA using the PrimeScript RT reagent kit (Takara), according to the manufacturer's instructions. RNA quality was first checked by measuring A_260_/A_280_ and A_260_/A_230_ and then assessed by gel electrophoresis, before performing quantitative real‐time PCR. The primers were designed using Primer Express v3.0 (Applied Biosystems, Foster City, CA, USA): 35 cycles were run with denaturation at 95°C for 15 s, annealing at 55°C for 30 s, and extension at 60°C for 45 s. The 16S rRNA gene was used as a control for standardization. Eight reported functional genes coding for QS regulation activity were chosen for these PCR analyses. Their corresponding primers are listed in Table S2. A melt‐curve analysis was performed to validate the specificity of the qRT‐PCR reaction. The relative transcription level of each gene was defined as the ratio of its transcripts obtained in biofilms grown under a given concentration of compounds over those obtained in LB medium with methanol, for which the $2^{-\Delta \Delta C_{t}}$ 2^−∆∆Ct^ method was applied (Livak and Schmittgen, 2001).

### Statistical analysis

Differences between means were determined through one‐way analysis of variance (ANOVA), with a *P* < 0.05 or *P* < 0.01 significance level. All data analyses were performed in SPSS software v13.0 (IBM Corporation, Armonk, NY, USA).

## Conflict of interest

None declared.

## Supporting information


**Fig. S1**. Effects of H12 extract on the dispersion of *P. aeruginosa* PAO1 biofilms.
**Fig. S2**. The *P. aeruginosa* PAO1 Biofilm attachment assay.
**Figure S3**. Confocal laser scanning microscopy (CLSM) photomicrographs of the PAO1 biofilm architecture, in the presence or absence of the QSI extract.
**Table S1.** Taxonomical identification and activity record of selected coral symbiotic bacteria.
**Table S2.** Primers used for the quantitative reverse transcriptase‐PCR amplified genes.
