# Supplementary material for: Antibiofilm activity substances derived from coral symbiotic bacterial extract inhibit biofouling by the model strain Pseudomonas aeruginosa PAO1
Source: Microb Biotechnol. 2018 Oct 9;11(6):1090–105. doi: 10.1111/1751-7915.13312 (PMC6196393; doi:10.1111/1751-7915.13312)

**Supporting information**

**Table S1.** Taxonomical identification and activity record of selected coral symbiotic bacteria. Note: We screened a total of 30 potential anti-QS isolates from the 200 culturable strains. After 16s-sequencing, five poor quality sequences were removed, leaving 25 high quality sequences that were BLAST-searched in the NCBI databases; after removing the duplicates, five bacteria were successfully identified (as shown in this table).

| Strain numbers | Species | Identity (%) | Anti-QS activity |
| --- | --- | --- | --- |
| H12, H5 and H33 | *Vibrio alginolyticus* | 100 | +++ |
| D11 and H8 | *Staphylococcus hominis* | 100 | +++ |
| H1, D2, D7, D8, H4, H6, H31, and H32 | *Lysinibacillus fusiform* | 99 | + |
| D12, D3, H3, H9, and H6 | *Bacillus cereus* | 99 | ++ |
| D35, D1, D9, D10, D26, H7, and H11 | *Staphylococcus warneri* | 99 | + |
| D4, D5, D6, H19, and H23 | Filtered poor quality sequences | NA | NA |

**Table S2.** Primers used for the quantitative reverse transcriptase-PCR amplified genes.

| Genes name | Primer sequence（F, 5'-3'） | Primer sequence（R, 5'-3'） |
| --- | --- | --- |
| *lasI* | CGCACATCTGGGAACTCA | CGGCACGGATCATCATCT |
| *lasR* | CTGTGGATGCTCAAGGACTAC | AACTGGTCTTGCCGATGG |
| *lasA* | CGCTGAATGACGACCTGTT | CTTTCGGGTTGATGCTGTAGT |
| *lasB* | GGTAGAACGCACGGTTGT | GGCAAGAACGACTTCCTGAT |
| *rhlR* | GCCAGCGTCTTGTTCGG | CGGTCTGCCTGAGCCATC |
| *rhlI* | GTAGCGGGTTTGCGGATG | CGGCATCAGGTCTTCATCG |
| *pqsA* | GACCGGCTGTATTCGATTC | GCTGAACCAGGGAAAGAAC |
| *pqsR* | CTGATCTGCCGGTAATTGG | ATCGACGAGGAACTGAAGA |

**Figure S1**. Effects of H12 extract on the dispersion of *P. aeruginosa* PAO1 biofilms. 24 hour *P. aeruginosa* PAO1 biofilms were exposed to different levels (1, 5, and 10 μg/mL) of H12 extract for 24 h. The remaining biofilm mass (A) and bacterial counts (B) were quantified. The data show the average value of three independent experiments performed in duplicate. Values are shown as the mean ± standard error. *P<0.05 compared with the negative control group.


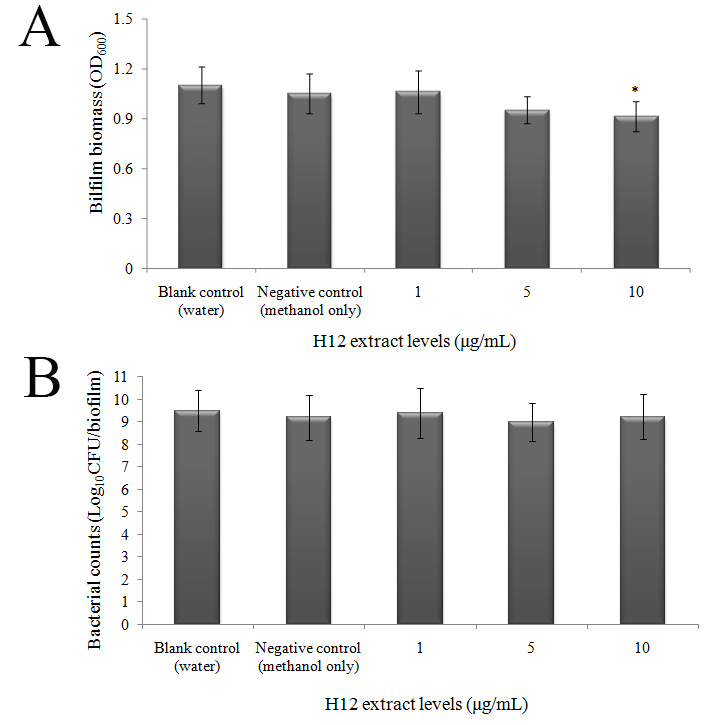


**Figure S2**. The *P. aeruginosa* PAO1 Biofilm attachment assay. The 1^#^, 2^#^, and 3^#^ represent the H12 extract at 1, 5 and 10 μg/ml level, respectively. The 4^#^ was the control (solvent carrier, methanol).


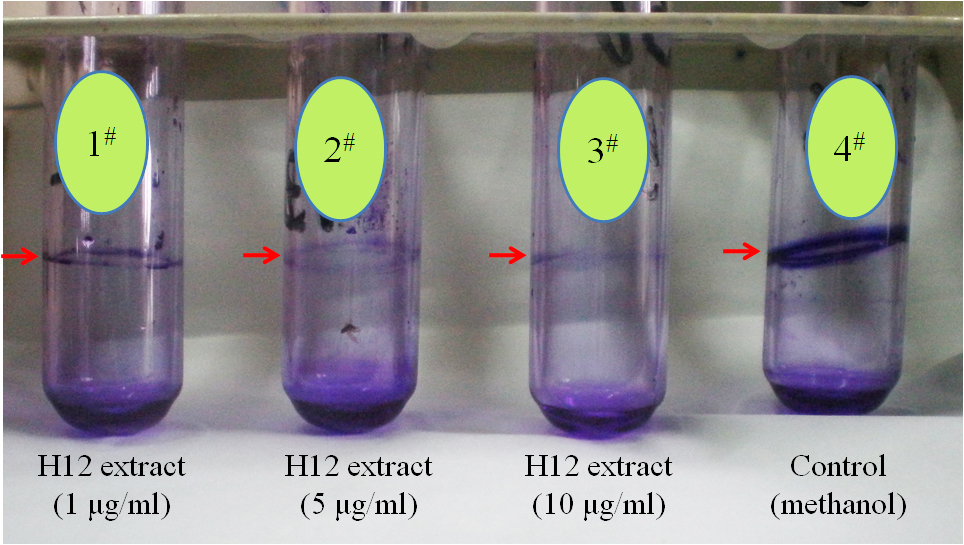


**Figure S3**. Confocal laser scanning microscopy (CLSM) photomicrographs of the PAO1 biofilm architecture, in the presence or absence of the anti-QS extract. Magnification was 200×. The images shown represent CLSM z-stack 3-D images of *P. aeruginosa* biofilm grown in the presence of methanol (a1, a2), furanone (b1, b2), and H12 strain extract (c1, c2) in LB media. The number of 1 and 2 represent the results after 12 hours and 36 hours, respectively. At 12 h, the live bacterial cells were labeled with syto9, which produced green fluorescence; at 36 h, the dead cells were labeled with PI, which produced green and red fluorescence, respectively, finally resulting in yellow. These 3-D images of *P. aeruginosa* biofilms show that PAO1 formed thinner and looser biofilms after treatment with the anti-QS extract.


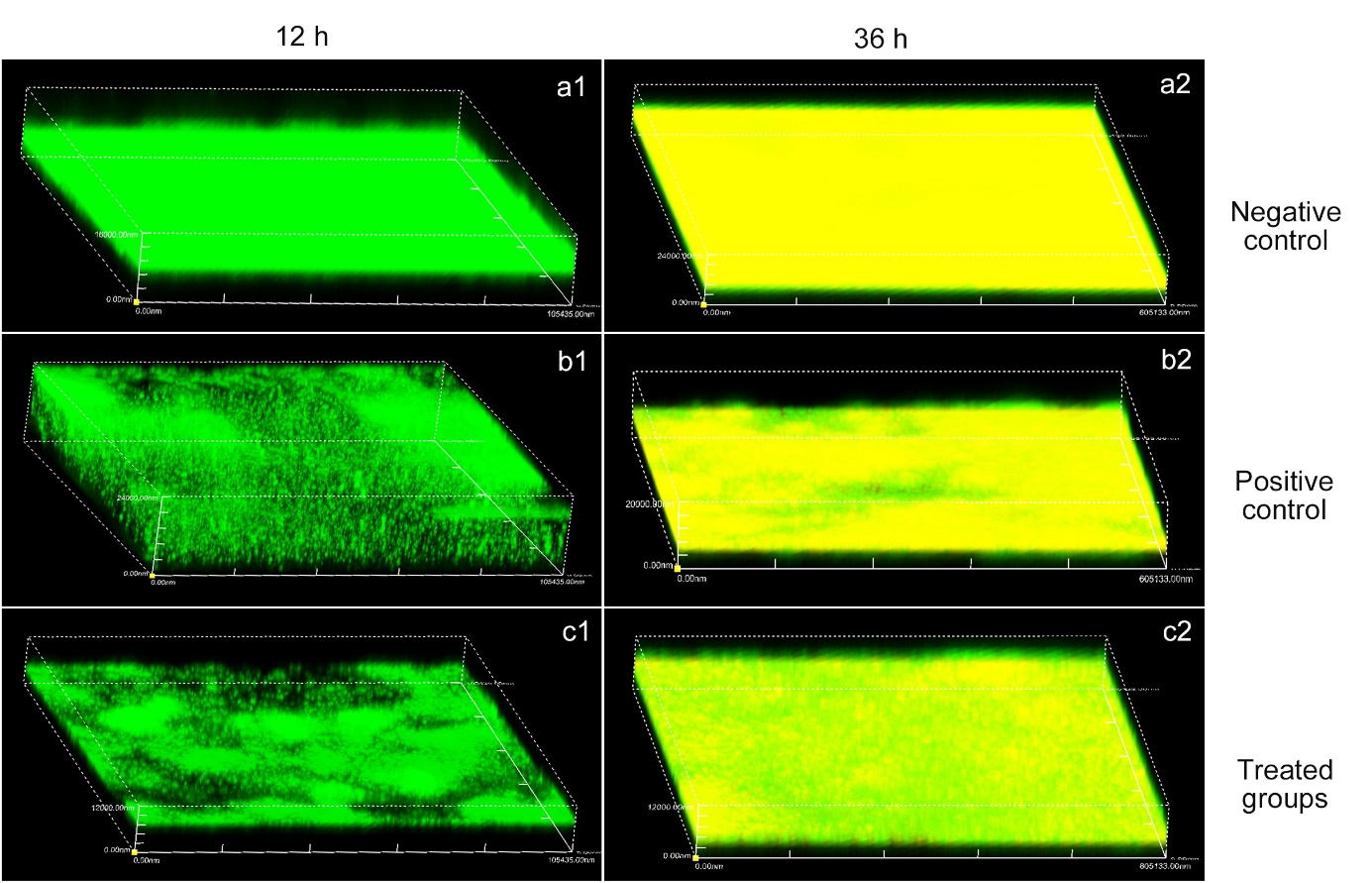

Supplement: Supplementary file 1 — Fig. S1. Effects of H12 extract on the dispersion of P. aeruginosa PAO1 biofilms. Fig. S2. The P. aeruginosa PAO1 Biofilm attachment assay. Figure S3. Confocal laser scanning microscopy (CLSM) photomicrographs of the PAO1 biofilm architecture, in the presence or absence of the QSI extract. Table S1. Taxonomical identification and activity record of selected coral symbiotic bacteria. Table S2. Primers used for the quantitative reverse transcriptase‐PCR amplified genes. [file MBT2-11-1090-s001.doc]
